# Banana Pseudostem Width Detection Based on Kinect V2 Depth Sensor

**DOI:** 10.1155/2022/3083647

**Published:** 2022-09-27

**Authors:** Jinzhi Wang, Xiuhua Li, Yonghua Zhou, Huaihai Wang, Minzan Li

**Affiliations:** ^1^College of Electrical Engineering, Guangxi University, Nanning 530004, China; ^2^State Grid Anhui Electric Power Co., Ltd., Feidong County Power Supply Company, Hefei 231600, China; ^3^Key Laboratory of Modern Precision Agriculture System Integration, Ministry of Education, China Agricultural University, Beijing 100083, China

## Abstract

This study used Kinect V2 sensor to collect the three-dimensional point cloud data of banana pseudostem and developed an automatic measurement method of banana pseudostem width. The banana plant was selected as the research object in a banana plantation in Fusui, Guangxi. The mobile measurement of banana pseudostem was carried out at a distance of 1 m from the banana plant using the field operation platform with Kinect V2 as the collection equipment. To eliminate the background data and improve the processing speed, a cascade classifier was used to recognize banana pseudostems from the depth image, extract the region of interest (ROI), and transform the ROI into a color point cloud combined with the color image; secondly, the point cloud was sparse by down-sampling; then, the point cloud noise was removed according to the classification of large-scale and small-scale noise; finally, the stem point cloud was segmented along the *y*-axis, and the difference between the maximum and minimum values in the *x*-axis direction of each segment was calculated as its horizontal width. The center point of each segment point cloud was used to fit the slope of the stem centerline, and the average horizontal width was corrected to the stem diameter. The test results show that the average measurement error is only 2.7 mm, the average relative error was 1.34%, and the measurement time is only about 300 ms. It could provide an effective solution for the automatic and rapid measurement of stem width of banana plants and other similar plants.

## 1. Introduction

Banana is one of the four largest fruits in the world and occupies an extremely important position in the world [1]. However, the management of banana plantation is still extensive. If the plant phenotype of banana plants can be extracted and applied to farm management, it will be of great significance for banana production efficiency and yield. The main body of banana plant is mainly composed of corms (true stems), pseudostems, leaves, and roots. Pseudostems mainly transport and store nutrients for leaves and fruits. The stem width or diameter of pseudostems largely determines the nutrient transport and supply capacity of the plant, and it is also related to the yield of the plant. Therefore, this study takes the pseudostem of banana plant as the measurement object and studies a rapid, automatic, and accurate method to measure the width of banana pseudostem, so as to guide scientific planting.

For the width measurement of banana pseudostem, the traditional method is generally manual measurement. The tools used include ruler, hand-held laser rangefinder and other tools, or equipment, which not only contains certain subjective factors but also takes time and effort. With the continuous development of technology, binocular vision, depth camera, laser scanning, 2D/3D LIDAR, CT, MRI, and other measurement technologies emerge endlessly. Because these technologies have the advantages of accuracy, speed, and low labor cost, they are widely used in the study of crop phenotypic feature extraction. Although laser scanners, CT, MRI, and other equipment have high advantages in accuracy or penetrability, they are also expensive; comparatively speaking, low-cost three-dimensional imaging devices such as binocular vision and depth sensor have also attracted more and more attention in agricultural research scenes such as fruit recognition and field plant phenotype measurement [2]. Researchers at home and abroad have also carried out a lot of research on low-cost three-dimensional imaging devices.

In recent 20 years, binocular vision based on stereo matching algorithm has been widely used in fruit recognition [3–5] and agricultural machinery navigation [6–8]. However, binocular vision has the problems of weak anti-interference, low matching accuracy, complex algorithm, and slow processing speed in the complex field environment, and there are some limitations and deficiencies in its application.

Based on the principle of laser ranging, Kinect V2 sensor can quickly obtain the color and three-dimensional point cloud data of the measured object. Compared with binocular vision, the Kinect sensor has low cost and strong resistance to environmental interference. At present, many researchers use it in the agricultural field to obtain plant phenotypic parameters and detect agricultural products [9–11]. Adar et al.[12] compared the current low-cost 3D imaging systems and concluded that the low-cost 3D imaging equipment can replace the laser scanner in many plant phenotype analysis scenes. Yamamoto et al. [13] designed a three-dimensional reconstruction method of apple using the Kinect depth sensor and estimated the fruit volume. Bao et al. [14] developed a noncontact automatic 3D robot blade detection system, which uses Kinect V2 sensor, high-precision 2D laser profiler, and six-axis robot manipulator to realize the automation of blade detection tasks. Hu et al. [15] proposed a nondestructive automatic growth measurement system for leafy vegetables based on Kinect. The system was used to obtain the precise three-dimensional model of the tested plants, and the key phenotypic parameters of the plants were measured according to the acquired model.

Because the banana plant is tall, the pseudostem is similar to the trunk, and the diameter is also large; the required measurement accuracy is not harsh, so it is very suitable to use low-cost Kinect series sensors for measurement. Therefore, in this study, Kinect V2 was used to obtain the three-dimensional point cloud data of banana pseudostems, and a point cloud data analysis and processing method were proposed to quickly calculate the pseudostem stem width, providing support for further prediction of banana crop growth and yield.

## 2. Materials and Methods

### 2.1. Experimental Data Collection

**Figure 1:**
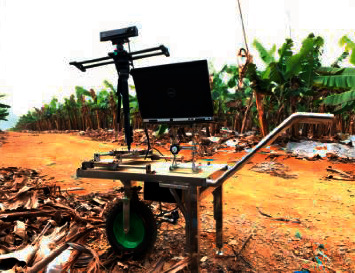
Field operation platform.

The image acquisition site of banana pseudostem point cloud is located in a banana plantation covering an area of 1800 mu in Guangxi subtropical agricultural science new city in Fusui County. During the experiment, it is in the fruit development period of banana. The spacing between each plant is about 0.5 m, and the spacing between rows is about 2 m. Data collection is carried out using the vehicle mounted field operation platform (as shown in Figure 1) composed of Kinect V2 sensor, Dell Precision 7530 Mobile Workstation (Intel-i9 CPU, 32 GB high-speed memory, NVIDIA QuADro P2000 graphics card), 220 V portable emergency energy storage power supply (2 pieces), and Beno KH25 tripod. Main parameters of Kinect V2 sensor are shown in Table 1.

**Figure 2:**
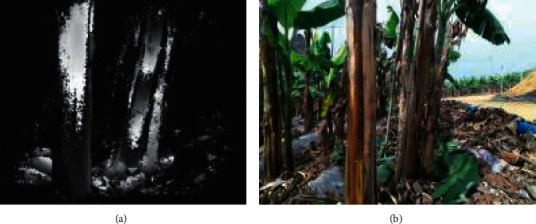
Images obtained by Kinect V2. (a) Depth image. (b) RGB image.

The shooting time of the experiment is from 9 a.m. to 5 p.m. By installing Kinect V2 on the vehicle mobile platform through a tripod, and moving at a speed of 8 m/s at a distance of 1.5 m from the ground height and 1 m from the banana pseudostem, the Kinect V2 is always aligned with the center of the banana pseudostem at a distance of 1 m by adjusting the wheel direction of the operation platform, to obtain the depth image and color image of the banana pseudostem. Five rows of banana plants were selected for mobile measurement. Some banana plants were randomly selected, and the resolution was 0 A. 01 mm digital vernier caliper was used to manually measure the diameter of the pseudostem at a height of 1 m. The gray value of each pixel in the depth image (Figure 2(a)) represents the linear distance from the measured point to the sensor. The color image (Figure 2(b)) was captured by the Kinect V2 internal integrated color camera.

### 2.2. Point Cloud Data Preprocessing

**Figure 3:**
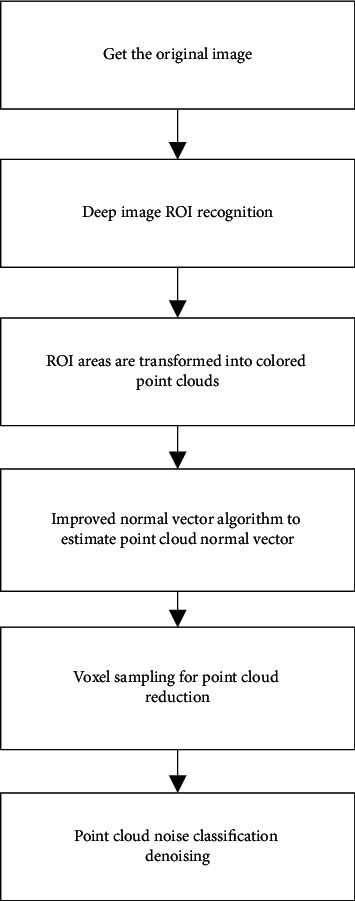
Point cloud pretreatment flow chart.

Before banana pseudostem estimation, the original data need to be preprocessed in order to effectively extract the measured banana pseudostem, reduce the measurement error, and improve the measurement speed. Preprocessing mainly includes four steps: ROI recognition, ROI extraction, conversion to point cloud, normal vector correction, voxel down-sampling, and point cloud filtering. The corresponding pretreatment process is shown in Figure 3.

#### 2.2.1. ROI Extraction from Depth Images Based on Cascaded Classifiers

**Figure 4:**
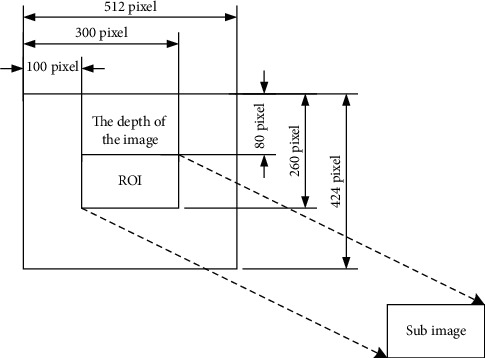
Schematic diagram of depth image ROI extraction.

Other background information in the collected original image, such as non-measured banana plants and fallen leaves, will interfere with the measurement. At the same time, the accuracy of the edge region of the depth image is also relatively low. At the same time, if all the information in the original data is transformed into color point cloud, and then the banana pseudostem is extracted by conditional filtering, a large amount of unnecessary information will be introduced, resulting in too long processing time and affecting the real-time performance. Therefore, the region of interest (ROI) of banana pseudostem can be extracted from the depth image. Only ROI part is converted to color point cloud, as shown in Figure 4. This method can effectively remove the background information and has good real-time performance with short processing time.

Considering that the angle, orientation, and distance of each shot are not fixed, the range of ROI is not fixed. Therefore, it is necessary to recognize the collected depth image and determine the ROI range containing banana pseudostems. In this study, 500 banana pseudostem depth image samples were randomly classified into training set and test set, including 250 training set and 250 test set samples. In this study, the banana pseudostem model is trained and recognized by cascade classification. The training samples include 250 positive samples and 500 negative samples. The positive samples are the depth images of banana pseudostems obtained by cutting the training and samples, and the negative samples are the depth images with the main body as the background obtained by random cutting. Because the pseudostems of banana crops are cylindrical and tightly wrapped by leaf sheaths, their toughness is poor, and their shape is usually straight. Although they are prone to slight inclination, they rarely bend significantly. Therefore, rectangles can be used to approximate the main body of pseudostems; both positive and negative samples are intercepted by a rectangle with an aspect ratio of 2 : 1. The classifier tool in OpenCV is used to train the positive and negative samples.

**Figure 5:**
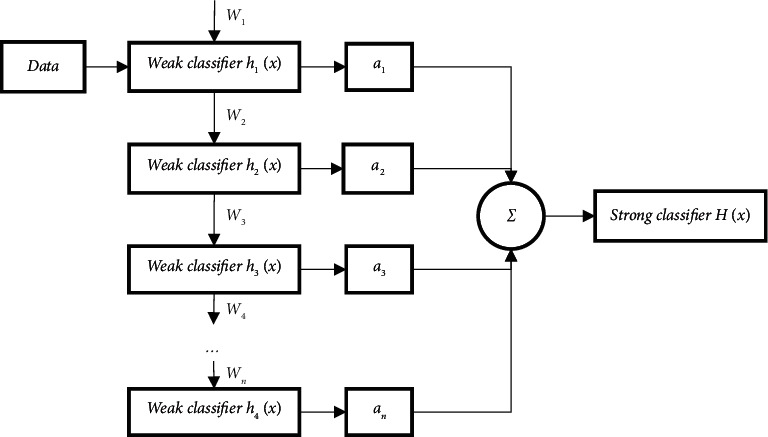
Strong classifier training process.

In the training process, the classifier will first extract the LBP features of the disparity map of the training samples. LBP is a feature that describes the local texture of an image and has the advantages of rotation invariance and gray invariance [16]. LBP operator is defined in 3×. In the window of 3, the central pixel is compared with the surrounding 8 pixels. The value of the pixel greater than the central pixel is assigned as 1, and the value of the pixel less than the central pixel is assigned as 0, to generate a new 8-bit binary number, and then it is converted it into a decimal number to replace the central pixel value. The training process of strong classifier is shown in Figure 4. First, the data and data weight *wi* are used to train the weak classifier, and one weak classifier is trained in each iteration. The trained weak classifier continues to participate in the next iteration. The weak classifier can be regarded as a feature trainer of LBP. Each simple feature corresponds to a weak classifier. After *n* weak classifiers are obtained, a strong classifier can be obtained by taking sign after linear combination through formula (1) [17]. The strong classifier training process schematic diagram is shown in Figure 5.

(1) $$\begin{array}{c} H\left(x\right)=\text{sign}\left[\overset{T}{\underset{t=1}{\sum }}\alpha_{t}h_{t}\left(x\right)\right]\text{,} \end{array}$$

where *t* is the sequence number of the weak classifier; *X* is LBP characteristic.

**Figure 6:**
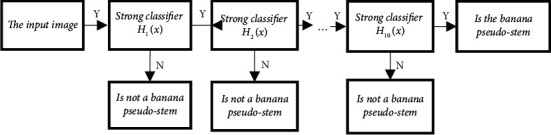
Cascade classifier flowchart.

**Figure 7:**
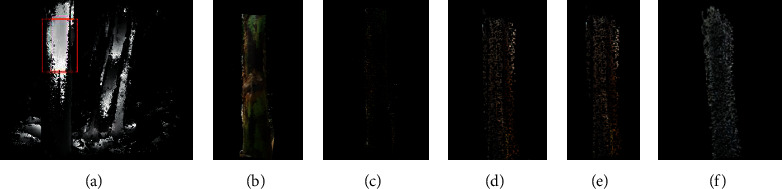
Results of the point cloud for each pretreatment step. (a) ROI recognition. (b) To a point cloud. (c) Voxel down-sampling. (d) Statistical filtering. (e) Radius filtering. (f) Bilateral filtering.

Furthermore, several strong classifiers are concatenated to get the final cascade classifier model. The cascade classifier trained in this study contains 10 strong classifiers, and its model structure is shown in Figure 6. The images to be tested must all meet the features of 10 strong classifiers in turn before they can be identified as banana pseudostems. The banana pseudostems are marked, and the corresponding ROI boundary is obtained. The identified ROI results are shown in Figure7(a). The recognition rate of banana pseudostem by this method is 92.3%, and the accuracy can be further improved if the number of effective samples is increased in the later stage.

#### 2.2.2. Convert Depth Image to Color Point Cloud

**Figure 8:**
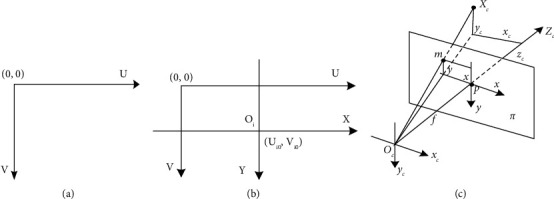
Coordinate transformation relationship. (a) Pixel coordinate system 7. (b) Image coordinate system 7. (c) Spatial coordinate system.

The depth image belongs to the pixel coordinate system, as shown in Figure 8(a). Each point in the image is represented by (*Ui*, *Vi*). Since the coordinates in the pixel coordinate system only contain the row and column information of the pixel, it has no physical meaning. Therefore, this study also needs to establish an image coordinate system *X*-*Y* with the meaning of actual physical dimensions, as shown in Figure 8(b).

Let the origin coordinate in the image coordinate system be *Oi*, and the coordinates in the pixel coordinate system be (*U*0, *V*0). *dx* and *dy* represent the actual physical size of each pixel. The *x-* and *y* -axes are parallel to the *u-* and *v* -axes, respectively. The pixel coordinate system is converted to the image coordinate system by the following formula:

(2) $$\begin{array}{c} \left[\begin{array}{c} x\\ y\\ 1 \end{array}\right]=\left[\begin{array}{ccc} dx & 0 & {-u}_{0}dx\\ 0 & dy & {-v}_{0}dy\\ 0 & 0 & 1 \end{array}\right]\left[\begin{array}{c} u\\ v\\ 1 \end{array}\right]\text{.} \end{array}$$

The image coordinate system is converted to the spatial coordinate system corresponding to Kinect V2, as shown in Figure 8(c).

The midpoint *m* in the figure is an imaging point in the image coordinate system, and the corresponding coordinates are (*x*, *y*). Then, the coordinate point m' in the corresponding spatial coordinate system is (*x*_*c*_, *y*_*c*_, *z*_*c*_), *f* is the focal length of the camera, and the depth value of point *m* is *d*. According to the principle of similar triangles, the following formula can be obtained:

(3) $$\begin{array}{c} \frac{f}{z_{c}}=\frac{x}{x_{c}}\\ =\frac{y}{y_{c}}\text{.} \end{array}$$

Then, it can be deduced that

(4) $$\begin{array}{c} \left[\begin{array}{c} x_{c}\\ y_{c}\\ z_{c} \end{array}\right]=\left[\begin{array}{ccc} \frac{z_{c}}{f} & 0 & 0\\ 0 & \frac{z_{c}}{f} & 0\\ 0 & 0 & z_{c} \end{array}\right]\left[\begin{array}{c} x\\ y\\ 1 \end{array}\right]\text{.} \end{array}$$

So far, the conversion relationship between the image coordinate system and the spatial coordinate system has been established. Through equations (2) and (4), the conversion relationship between the pixel coordinate system and the spatial coordinate system can be obtained as follows:

(5) $$\begin{array}{c} \left[\begin{array}{c} x_{c}\\ y_{c}\\ z_{c} \end{array}\right]=\left[\begin{array}{ccc} \frac{z_{c}}{f} & 0 & 0\\ 0 & \frac{z_{c}}{f} & 0\\ 0 & 0 & z_{c} \end{array}\right]\left[\begin{array}{ccc} dx & 0 & {-u}_{0}dx\\ 0 & dy & {-v}_{0}dy\\ 0 & 0 & 1 \end{array}\right]\left[\begin{array}{c} u\\ v\\ 1 \end{array}\right]\text{.} \end{array}$$

Therefore, the ROI extracted from the depth image can be transformed into the spatial coordinate system by equation (5). Since Kinect V2 has been calibrated before leaving the site, internal parameters can be called through API, and color point cloud can be generated through PCL in combination with color information. The result of converting ROI to point cloud is shown in Figure 7(b).

#### 2.2.3. Point Cloud Normal Vector Correction

As a basic morphological feature of point cloud, the quality of normal vector will have a significant impact on the subsequent point cloud dilution, point cloud smoothing, and point cloud computing. At present, the commonly used point cloud normal vector estimation method is the key component analysis method based on point cloud local covariance analysis. *V* is set as the whole point cloud set, and a point in the point cloud *m*_*i*_ ∈ *V* and its set of *k* nearest neighbors (hereinafter referred to as *K* neighborhood) *N*(*m*_*j*_) are given, and the following covariance matrix *C* can be constructed:

(6) $$\begin{array}{c} C=\frac{1}{k}\underset{m_{i}\in N\left(m_{j}\right)}{\sum }{\left(m_{i}-\bar{m}\right)\left(m_{i}-\bar{m}\right)}^{T}\text{,} \end{array}$$

where $\bar{m}=1/k\sum_{i=1}^{k}m_{i}$ is the centroid of the neighborhood of *m*_*i*_ point *K.*

**Figure 9:**
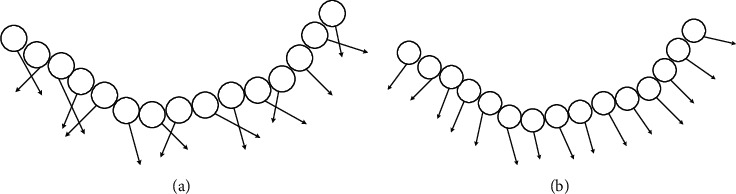
Schematic diagram of normal vectors before and after improvement. (a) Before improvement. (b) After improvement.

Through eigen root decomposition of the covariance matrix *C*, the eigenvector of the corresponding minimum eigen root is the approximate value of the normal phasor at point *MI*. Although the covariance analysis method has certain anti-interference ability, its anti-interference ability decreases obviously when the point cloud noise is too complex. Therefore, the Gaussian weight function method is added to the original covariance matrix to smooth the vector, and the following expression is obtained.

(7) $$\begin{array}{c} C=\frac{1}{k}\underset{m_{i}\in N\left(m_{j}\right)}{\sum }{e^{{-||m_{i}-\bar{m}||}^{2}/\sigma^{2}}\left(m_{i}-\bar{m}\right)\left(m_{i}-\bar{m}\right)}^{T}\text{,} \end{array}$$

where ||·|| represents the modulus of a vector and *σ* is a point cloud density parameter, and the corrected normal vector information can be obtained by eigenvalue decomposition. Normal vectors before and after improvement are shown in Figure 9.

Kinect V2 is used to collect the wall point cloud, and the traditional normal vector estimation method and the improved normal vector estimation method are used to estimate the normal vector, respectively. Through observation, it is found that the improved normal vector becomes more uniform on the point cloud model, and the divergence direction tends to be more consistent, which makes the subsequent down-sampling and bilateral filtering process more efficient and accurate.

#### 2.2.4. Voxel Down-Sampling

Because this study mainly measures the stem width of banana pseudostem, too dense point cloud will reduce the measurement accuracy and real-time performance. Therefore, this study carries out voxel down-sampling on the point cloud to achieve point cloud dilution. Voxel refers to a three-dimensional image with a side length of *λ* pixel cube, and voxel downsampling is mainly based on the side length *λ*. Voxel downsampling is mainly to decompose voxels into several small voxel grids with side length λ according to a certain ratio according to the size of side length λ/k. The improved normal vector estimation is carried out for each small voxel cube, and the corresponding center of gravity is estimated. Only the center of gravity and the nearest original point are retained, to approximately represent all points in the whole voxel. While protecting the detailed information of the point cloud, the point cloud is simplified. Through down-sampling, the number of point clouds decreased from 13655 to 3814, a decrease of 72%. The processed results are shown in Figure 7(c).

#### 2.2.5. Point Cloud Filtering

Due to the equipment itself, operator experience, measurement environment, and other factors, a lot of noise will be generated in the process of point cloud data acquisition. Therefore, it is necessary to filter the point cloud before measurement. According to the specific situation of this study, this study proposes a method of noise reduction by classification of large-scale and small-scale noise. Large-scale noise refers to the small and dense point cloud, which is far away from the center of the main point cloud and the sparse points, which are suspended above the main point cloud and deviate from the main point cloud. It has the characteristics of large amplitude and high frequency. Small size noise refers to some irregular data points entangled with the main point cloud. Compared with the single filtering method, the classification noise reduction method used in this study can achieve better noise reduction effect.

*(1) Large Size Noise Removal.* In this study, the combination of statistical filtering and radius filtering is used to remove large-scale noise. Statistical filtering [16] means that for any point, the average distance between the point and other points in the *k* field is calculated, and the distribution of the results is assumed to follow the Gaussian distribution. Calculate the mean *μ*and standard deviation *σ* of the distance between this point and its points in the neighborhood of *K*.Keep the points that are within the range of (*μ* − *σ*, *μ* + *σ*). Radius filtering means that for a certain subject point *p* in the point cloud data, it is considered that at least *M* points should exist in the neighborhood with radius *r* of the subject point; otherwise, it will be determined as discrete points and deleted. The corresponding statistical filtering and radius filtering effects are shown in Figures 7(d) and 7(e).

*(2) Small Size Noise Smoothing.* Bilateral filtering is a common method in image filtering, which has been extended to 3D point cloud data model filtering. The bilateral filtering of 3D point cloud data model is to move the noise points along the direction of their normal vector and constantly adjust the position and coordinates of the noise points to smooth the small-scale noise [18].

The expression of bilateral filtering is shown as follows:

(8) $$\begin{array}{c} p_{i}^{'}=p_{i}+\alpha \cdot n\text{,} \end{array}$$

where *p*′_*i*_ is the filtered point, *p*_*i*_ is the original data point, *α* is the bilateral filter factor (as shown in (9)), and *n* is the normal vector of point *p*_*i*_.

(9) $$\begin{array}{c} \alpha =\frac{\underset{p_{j}\in N\left(p_{i}\right)}{\sum }W_{c}\left(||p_{i}-p_{j}||\right)W_{s}\left(||\langle n_{i},n_{j}\rangle -1||\right)\langle p_{i}-p_{j},n_{j}\rangle }{\underset{p_{j}\in N\left(p_{i}\right)}{\sum }W_{c}\left(||p_{i}-p_{j}||\right)W_{s}\left(||\langle n_{i},n_{j}\rangle -1||\right)}\text{.} \end{array}$$

In equation (9), *p*_*j*_ is the neighborhood point of the data point *p*_*i*_; ||·||represents the module of the vector; 〈∙〉 represents the inner product of the vector; *n*_*i*_ is the normal vector of point *p*_*i*_ on the point cloud; and *n*_*j*_ is the normal vector of the adjacent point *p*. *W*_*c*_(*x*) and *W*_*s*_(*y*) are smoothing filter weight function (equation (10))and feature preserving weight function (equation (11)), respectively; *σ*_*c*_ and *σ*_*s*_ represent the filtering parameters in the Gaussian weight function, which reflects the influence range of tangent and normal vectors when calculating the bilateral filtering factor of any sampling point.

(10) $$\begin{array}{c} W_{c}\left(x\right)=e^{\left[-x^{2}/\left(2\sigma_{c}^{2}\right)\right]}\text{,} \end{array}$$

(11) $$\begin{array}{c} W_{s}\left(y\right)=e^{\left[-y^{2}/\left(2\sigma_{s}^{2}\right)\right]}\text{.} \end{array}$$

The bilateral filtering process is as follows:

1. For point cloud data points, the *k* neighborhood is determined by *p*_*i*_, and the normal vectors corresponding to all points in the neighborhood are solved by the improved normal vector method.
2. The smoothing filter function *W*_*c*_(*x*) parameter *x*=||*p*_*i*_ − *p*_*j*_|| of data point *p*_*i*_ (distance between data point *p*_*i*_ and *p*_*j*_) and feature retention weight function *W*_*s*_(*y*) parameter *y*=||〈*n*_*i*_, *n*_*j*_〉 − 1|| (the inner product of the angle between the normal vector between point *p*_*i*_ and *p*_*j*_) are solved. Combined with equations (9)–(11), the improved bilateral filter factor is solved *α*.
3. The new coordinate *p*_*i*_ of the data point *p*_*I*_′ is calculated according to equation (8), and the point *p*_*i*_ is moved to *p*_*I*_′ coordinate.

The processed image is shown in Figure 7(f).

### 2.3. Estimation of Banana Pseudostem Width

**Figure 10:**
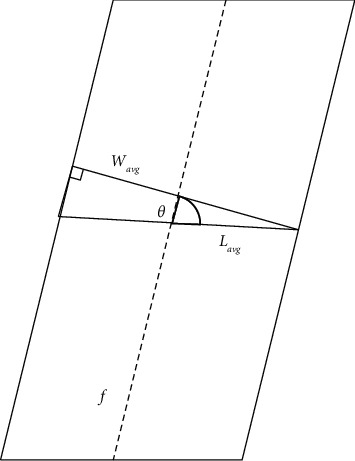
Schematic diagram of angle correction.

Because the banana pseudostem is thick at the bottom and thin at the top, to reduce the measurement error, this study evenly segments the pretreated point cloud from top to bottom, measures the width of each segment of point cloud, sets the condition threshold for judgment and angle correction, and finally takes the average value to obtain the measurement results. The specific algorithm flow is as follows:

(1) The maximum and minimum value points of the pretreated point cloud in the *y*-axis (height) direction are searched and marked as *Y*_max_ and *Y*_min_.
(2) The point cloud is divided into *n* segments from bottom to top along the *y*-axis.
(3) Each segmented point cloud is queried separately, the maximum value point and minimum value point in the *x*-axis (width) direction are searched, and they are recorded as (*X*_*i*_ max_, *Y*_*i*_ max_) and (*X*_*i*_ min_, *Y*_*i*_ min_). It can be obtained that the width *L*_*i*_ of the point cloud of segment *i* in the *x*-axis direction is as follows:
  (12) $$\begin{array}{c} L_{i}=X_{i\_max}-X_{i\_min}\text{.} \end{array}$$
(4) The width of pseudostem is easily affected by withered leaves on the stem, resulting in great changes in the width of some segments. To filter out this effect, the banana pseudostem range thresholds *L*_max_ and *L*_min_ and the banana pseudostem offset threshold *δ* are set. Whether *L*_*i*_ satisfies equation (13) at the same time is judged, and if so, it will be recorded as the effective value. This segment of point cloud is called the effective point cloud. By traversing *L*_*i*_, we get *N* valid values
  (13) $$\begin{array}{c} \left\{\begin{array}{c} {L_{\min}\leq L}_{i}\leq L_{\max}\\ \left|L_{i}-\left.L_{i-1}\right|\leq \delta \right.\\ \left|L_{i}-\left.L_{i+1}\right|\leq \right.\delta \end{array}\right.\text{.} \end{array}$$
(5) The average value *L*_avg_ of *n* effective point cloud widths *L*_*i*_ is found as the average width in the horizontal direction of banana stem.
(6) Since banana pseudostems are inclined to some extent during the growth process, the horizontal span value of each stem point cloud calculated in the previous step is not equal to the diameter of the stem, so the width obtained in step 5 needs to be corrected (see Figure 10 for the schematic diagram). First, the midpoint coordinates (*X*_*i*_*mid*_, *Y*_*i*_*mid*_) of each effective point cloud on the *X-* and *Y*-axes according to equation (14)are calculated; The center line *f* of the stem was fitted according to the midpoint coordinates of *N* effective point clouds, and the inclination Angle θ between the center line *f* and the X-axis was calculated. The tilt Angle θ of center line *f* is the tilt of banana pseudostem finally, the vertical section width *W*_avg_ of the stem is calculated by equation (15).
  (14) $$\begin{array}{c} \left\{\begin{array}{c} X_{i\_mid}=\frac{\left(X_{i\_max}+X_{i\_min}\right)}{2}\\ Y_{i\_mid}=\frac{\left(Y_{i\_max}+Y_{i\_min}\right)}{2} \end{array}\right.\text{.} \end{array}$$
  (15) $$\begin{array}{c} W_{avg}=L_{avg}\sin\;\theta \text{.} \end{array}$$

## 3. Results and Discussion

**Figure 11:**
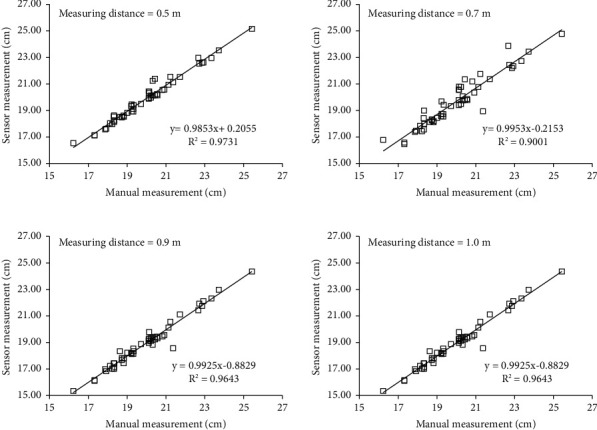
Result comparison between sensor and manual measurement.

The measurement results at different distances are shown in Table 2 and Figure 11. It can be seen that under different distances, the correlation coefficient *r* between the stem width measured by the sensor and the manual measurement results basically reaches more than 0.95. The lowest error occurs at a distance of 0.5 m, where the mean absolute error is 2.68 mm and the mean relative error is 1.34%; the overall error increases obviously with the increase in the measurement distance, and the average relative error at the distance of 1.0 m is as high as 8.35%. It shows that the measurement accuracy of Kinect V2 sensor is obviously affected by distance. In addition, it can be seen from the measurement results that the average value of the sensor decreases gradually with the increase in the distance; that is, compared with the average value of manual measurement, the farther the distance, the greater the negative measurement error of the sensor. This is mainly due to the thinning of the beam quantity projected by Kinect V2 onto the banana pseudostem, resulting in the measured value of the sensor being gradually lower than the manual measured value.

Through comprehensive analysis, the main causes of measurement errors are as follows: (1) the uneven surface of banana pseudostem leads to inevitable errors in manual measurement; (2) the structure of Kinect V2 sensor causes the measurement accuracy to decrease gradually with the increase in distance, resulting in systematic error. In addition, in terms of processing speed, ROI recognition time is 100 ms, point cloud conversion time is about 50 s, down-sampling time is about 50 ms, combined filtering time is about 80 ms, measurement time is about 100 ms, and the total measurement time is about 300 ms.

## 4. Conclusion

1. Compared with the method of converting the depth image into point cloud and then adjusting the *x-*, *y-,* and *z*-axes, the method of using cascade classifiers to recognize ROI from the depth image and then convert ROI into point cloud greatly shortens the measurement time and improves the measurement accuracy.
2. By improving the method of point cloud normal vector estimation, the normal vector obtained by estimation is more uniform and smooth, and the measurement accuracy is improved.
3. According to the difference in the size of point cloud noise, the point cloud is classified into large-scale and small-scale noise. The large-scale noise is removed by statistical filtering and radius filtering, and the small-scale noise is smoothed by bilateral filtering. This method can remove the noise more effectively by differentiating the point cloud noise. Comparing the data before and after the point cloud filtering, the average absolute error is reduced by 35.33 mm and the average relative error is reduced by 17.68% after the point cloud filtering at 0.5 m. The experimental results show that the combined filter has a good effect on noise removal.
4. According to the characteristics of banana stem, the stem point cloud is divided into *n* segments from bottom to top, and the span of each segment in the *x*-axis is obtained and averaged to reduce the measurement error; the stem width was corrected by fitting the stem centerline with the approximate center point to obtain the inclination angle of the stem. The average absolute error is 5.4 mm and the average relative error is 2.70% by comparing the data before the angle correction with the manual measurement data at 0.5 m; the measurement accuracy is improved by 2.7 mm after comparison and correction. At 0.7 m, the average absolute error is 8.8 mm and the average relative error is 8.80% compared with the manual measurement data; the measurement accuracy is improved by 2.9 mm after comparison and correction. It is found that the angle correction is closer to the manual measurement value. The measurement method has high accuracy, can meet the needs of plant stem measurement under a reasonable measurement distance, and can provide technical support for the extraction of key growth parameters of banana plants.
5. The detection method proposed in this study is not only limited to the detection of banana false stem but also can be applied to the detection of tree stem and other fields. The experimental results show that the method has better measurement speed and accuracy. In the industrial field, the method can also quickly and accurately detect the width and diameter of columnar objects such as telegraph poles. However, the method adopted in this study also has some limitations. This method is not suitable for some small objects and some areas that need accurate measurement, which will lead to excessive relative errors. At the same time, due to the use of active light source to obtain data, it is not suitable for the measurement of transparent, translucent, and reflective objects, which will reduce the measurement accuracy in the strong lighting environment. [19–25].

## Figures and Tables

**Table 1:** Key parameters of Kinect V2 sensor.

Parameter	Value
RGB color image resolution	1920 × 1080
Depth (infrared) image resolution	512 × 424
Frame rate (FPS)	30 fps
Horizontal field angle	70 degrees
Vertical field angle	60 degrees
Detection range	0.5ཞ4.5 m

**Table 2:** Measurement results at different distances.

Measuring distance (m)	Measured average value (mm) (average value of manual measurement = 199.8 mm)	Correlation coefficient *r* between sensor and manual measurement results	Mean absolute error (mm)	Mean value of relative error (%)
0.5	198.9	0.987	2.7	1.34
0.7	196.7	0.949	5.9	2.89
0.9	189.5	0.982	10.3	5.25
1.0	183.8	0.977	16.0	8.35

## Data Availability

The dataset can be accessed upon request.
